# KIR3DL1 and HLA-Bw4 Interaction Showed a Favorable Role in Patients with Myelodysplastic Syndromes in Chinese Southern Han

**DOI:** 10.1155/2020/6215435

**Published:** 2020-04-29

**Authors:** Jian Xin Zhen, Si Qi Cai, Yuan Tao Chen, Zhi Chao Yang, Jia Cai Zhuo, Zhi Hui Deng

**Affiliations:** ^1^Central Laboratory, Shenzhen Baoan Women's and Children's Hospital, Shenzhen, Guangdong 518102, China; ^2^Immunogenetics Laboratory, Shenzhen Blood Center, Shenzhen, Guangdong 518035, China; ^3^Department of Biology, College of Arts & Sciences, University of Virginia, Charlottesville, VA 22904, USA; ^4^Department of Hematology, Shenzhen Second People's Hospital, Shenzhen, Guangdong 518034, China; ^5^Department of Transfusion Medicine, School of Laboratory Medicine and Biotechnology, Southern Medical University, Guangzhou, Guangdong 510515, China

## Abstract

**Background:**

The association studies of killer cell immunoglobulin-like receptors (KIRs) with the occurrence of myelodysplastic syndromes (MDS) are limited worldwide; this study investigated the genetic risk/protective factors of MDS in KIR and human leucocyte antigen (HLA) systems to gain a better understanding of the role played by KIR and their cognate HLA ligands in MDS pathogenesis.

**Methods:**

We genotyped a total number of 77 patients with MDS from Chinese Southern Han and 745 healthy controls for the *KIR* loci and *HLA class I*. The carrier frequencies of *KIR* genes, *KIR* genotypes, *class I HLA* ligands, and *KIR-HLA* combinations were calculated by direct counting. The effect of individual *KIR* genes and *HLA* ligands on MDS risk was evaluated by logistic regression analyses using SAS 9.2 software.

**Results:**

We found that neither the *KIR* genes nor the *KIR* genotypes were associated with the occurrence of MDS. However, we observed that the frequencies for the strong inhibitory ligand *HLA-Bw4* as well as *KIR3DL1-HLA-Bw4* combination were significantly higher in healthy controls than those in the MDS patient group, respectively (73.42% *vs.* 62.34%, *P* = 0.038; 70.87% *vs.* 59.74%, *P* = 0.043).

**Conclusion:**

Our results showed that HLA-Bw4 ligand and KIR3DL1-HLA-Bw4 combination could confer a protective effect against MDS in Chinese Southern Han.

## 1. Introduction

Natural killer (NK) cells are a key component of the innate immune system and play an important role in antitumor surveillance [1]. The response and function of NK cells are regulated by killer cell immunoglobulin-like receptors (KIRs) that are expressed on both NK cells and some T cell subsets. The *KIR* gene cluster on human chromosome 19 consists of 14 functional *KIR* genes (*KIR2DL1~5*, *2DS1~5*, *3DL1~3*, and *3DS1*) and two pseudogenes (*KIR2DP1* and *3DP1*) and inherited as haplotype *A* or *B*. The difference between them is that *KIR A* have a fix gene group of more inhibitory genes and only activating gene *KIR2DS4*, whereas haplotype *B* represents a diverse group of haplotypes with variable numbers of activating *KIR* genes [2].

The function of KIR on NK cells is dependent on the normal expression of class I HLA ligands on the target cell. The partnership between some of the inhibitory KIRs and their HLA ligands is well established. The two domain inhibitory KIRs KIR2DL1, 2DL2/3 bind HLA-C2 (C allotypes with lysine at residue 80) and HLA-C1 (C allotypes with asparagine at residue 80), respectively. KIR3DL1 recognizes HLA-A and HLA-B allotypes with the Bw4 motif (defined by residues 77-83), and Bw4 can be further divided into allotypes with isoleucine at position 80 (Bw4-80I) and those with threonine at this position (Bw4-80T). KIR3DL2 recognizes HLA-A3 and A11. Less is known about the activating KIR ligands, though KIR2DS1 and KIR2DS5 are known to bind C2^+^HLA-C, albeit with lower affinity than KIR2DL1 [3, 4].

In recent years, the clinical significance of the increasingly recognized *KIR* polymorphism in tumor, infectious disease, and transplantation has drawn extensive interest. Myelodysplastic syndromes (MDS), which were first described as “preleukemia” by Block et al. in 1953 [5], are a heterogeneous group of clonal hematopoietic stem cell malignancies characterized by ineffective hematopoiesis and peripheral blood cytopenias, and patients typically have a hypercellular bone marrow [6, 7]. Approximately 40% of MDS patients frequently evolve into acute myeloid leukemia (AML) [6, 7]. In our previous study, the decreased frequencies of *KIR AA* genotype were found in three subtypes of leukemia when compared to healthy controls: acute lymphoblastic leukemia (ALL, OR = 0.68, 95%CI = 0.52‐0.89, *P* = 0.004), AML (OR = 0.76, 95%CI = 0.59‐0.98, *P* = 0.034), and chronic myeloid leukemia (CML, OR = 0.72, 95%CI = 0.51‐1.01, ns). We also observed the similar trend for non-Hodgkin's lymphoma (NHL, OR = 0.47, 95%CI = 0.26‐0.88, *P* = 0.017), which implied that the *KIR AA* genotype could confer differential protection against ALL, AML, CML, and NHL in Chinese Southern Han [8]. Whether *KIR AA* genotype that carried more *KIR* inhibitory genes might play a similar role in immune surveillance of MDS? The present study investigated the frequencies of *KIR* genes, *KIR* genotypes, *class I HLA* ligands, and *KIR-HLA* combinations with an aim to provide clues for better understanding pathogenesis of MDS in Chinese Southern Han.

## 2. Materials and Methods

### 2.1. Study Subjects

This study consisted of 77 patients with MDS (mean age: 35.7, range 18-84 years) and 745 healthy unrelated individuals. Samples for patients with MDS were consecutively enrolled and randomly selected from cases for the hematopoietic stem cell transplantation (HSCT) program in the immunogenetics laboratory of Shenzhen Blood Center during the period of August 2008 to June 2018. Diagnosis of MDS followed the World Health Organization Classification [9, 10].

The healthy control group (*n* = 745) was recruited from blood volunteer donors with a mean age of 32.7 (range18-55 years) in Shenzhen Blood Center, China. The age range is equivalent to the case group. Written informed consent was obtained from each donor. 5 mL EDTA anticoagulated peripheral blood was collected and stored at -20°C.

### 2.2. Genomic DNA Isolation

Genomic DNA was prepared from peripheral blood using a MagCore Nudeic Acid Extractor (MagCore, Taiwan, China). DNA was extracted from 0.4 mL of whole blood anticoagulated with 5% EDTA. The concentration of each DNA sample was adjusted to 50-100 ng/*μ*L.

### 2.3. KIR Genotyping

The presence or absence of *KIR* genes was determined by using polymerase chain reaction with sequence-specific primer (PCR-SSP), using the KIR-Ready Gene kit (inno-train Diagnostik GmbH, Germany). The PCR products were electrophoresed on a 2% agarose gel and visualized under ultraviolet light. *KIR* haplotype *A* and *B* were defined based on the presence or absence of specific *KIR* genes, as previously described [2].

### 2.4. HLA High-Resolution Genotyping

*HLA-A*, *HLA*-*B*, and *HLA*-*C* high-resolution typing was performed by using the AlleleSEQR HLA SBT kit (Atria Genetics, San Francisco) following the instruction. Exons 2, 3, and 4 of *HLA-A*, *HLA*-*B*, and *HLA*-*C* were sequenced on an ABI 3730XL DNA sequencer (Applied Biosystems, Foster City, CA, USA). Exons 5 and 6 of *HLA-C* locus were also sequenced when necessary. Four-digit *HLA* genotypes were assigned using the ASSIGN 4.7 software (Conexio Genomics, Applecross, Australia). HLA-B and HLA-C allotypes were then grouped according to their KIR-binding motifs (HLA-Bw4 from HLA-Bw6 and HLA-C1 from HLA-C2).

### 2.5. Statistical Analysis

Carrier frequency for *HLA* ligands and *KIR* genes was calculated by direct counting. The effect of individual KIR and HLA ligands on disease outcome was evaluated by logistic regression analyses using SAS 9.2 (SAS Institute). PROC LOGISTIC was used to obtain odds ratios and the 95% confidence intervals. The statistical significance refers to two-sided *P* values of <0.05.

## 3. Results

### 3.1. Comparison Analysis for KIR Genes and Genotypes

**Figure 1:**
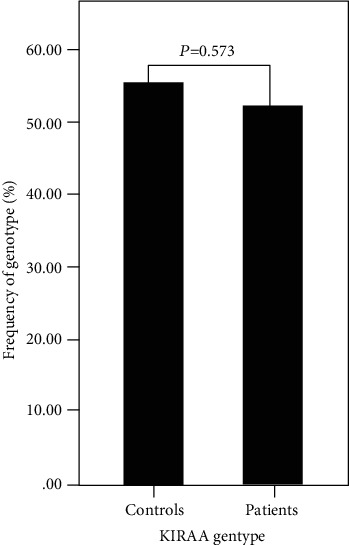
Comparison analysis for *KIR AA* genotype between MDS patients and controls, there was no significant difference (*P* > 0.05).

The presence of individual *KIR* genes did not associate significantly with risk of MDS. We further tested the distribution of the *KIR AA vs. Bx* genotypes (*x* represents haplotype *A* or *B*) in patients and controls; the frequency of *KIR AA* showed no significant difference between the healthy controls and the MDS patients (55.30% *vs.* 51.95%, *P* = 0.573; Table 1 and Figure 1).

### 3.2. Comparison Analysis for HLA Ligands and KIR/HLA Combinations

Understanding the role of KIR in MDS was also refined further by considering the class I HLA ligands for KIR in the analysis. There was no significant difference in the frequency distribution of *HLA-C1/C2* genotype. However, the frequencies of strong inhibitory ligand *HLA-Bw4* and *KIR3DL1-HLA-Bw4* combination were significantly higher in healthy controls than those in MDS patients, respectively (*HLA-Bw4*: 73.42% *vs.* 62.34%, *P* = 0.038; *KIR3DL1-HLA-Bw4*: 70.87% *vs.* 59.74%, *P* = 0.043). Dividing Bw4 allotypes into Bw4-80I and Bw4-80T, the significant differences remained in *HLA-Bw4-80I* and *KIR3DL1-HLA-Bw4-80I* (*HLA-Bw4-80I*: 54.23% *vs.* 40.26%, *P* = 0.019; *KIR3DL1-HLA-Bw4-80I*: 52.62% *vs.* 37.66%, *P* = 0.012), but not in *HLA-Bw4-80*T and *KIR3DL1-HLA-Bw4-80T*. No significant difference was observed for other *class I HLA* ligands or *KIR-HLA* combinations between MDS patients and healthy controls (Table 2).

## 4. Discussion

Besides an activated state of the adaptive immune system by increased percentages of activated T cells and decreased frequencies of regulatory T cells, innate immune responses play an important role in immune surveillance of MDS [11, 12]. Increased malignant clones and cellular immunodeficiency contribute to the pathogenesis of MDS. Cytotoxic T lymphocytes play a central role in antitumor immunity, but unfortunately often exhibit exhaustion induced by myeloid-derived suppressor cells in MDS patients through the TIM3/Gal-9 pathway [13]. NK cells mediate cytotoxicity of bone marrow precursor cells in low-risk MDS. Increased NK-cell frequencies were observed in low-risk MDS as compared to high-risk MDS [12]. Moreover, NK cells of MDS patients expressed increased levels of cytotoxic granules with granzyme B, the most important cytokines in NK cells against infectious and malignant cells [11]. Up to 90% autologous cytotoxicity against aberrant hematopoietic precursor cells was not HLA class I restricted but NK cell dependent [11]. NK cell-deficient patients were predominantly found in high-risk MDS, and deficiency of NK cells was strongly associated with poor prognosis [14].

Polymorphic inhibitory receptors educate NK cells, enabling them to kill tumors or infected cells having altered or reduced HLA class I expression [8]. In the present study, we found that the strong inhibitory HLA-Bw4 ligand and KIR3DL1-HLA-Bw4 combination could confer a protective effect against MDS in Chinese Southern Han; *KIR AA* genotype was not associated with the occurrence of MDS. In our previous Chinese leukemia research, *KIR AA* genotype appeared to protect against three major leukemia types, but HLA-Bw4 ligand and KIR3DL1-HLA-Bw4 combination were not associated with Chinese leukemia [8]. Interestingly, a recent study from Stringaris et al. demonstrated that *KIR* haplotype *A* was an independent risk factor for the progression of MDS to AML [15]; patients with haplotype *A* had worse adjusted progression-free survival (RR = 2.96, *P* = 0.001) and overall survival (RR = 2.25, *P* = 0.02) compared with *KIR* haplotype *B*, which seemed deceptively paradoxical to our present results. Unlike the study of Stringaris et al., the present study had not investigated the risk/protective factors for progression of MDS to AML but focused on the association of KIR receptors and their cognate ligands with the occurrence of MDS. The differential effect played by *KIR AA* genotype in the above three studies might be partly resulted from the distinct maturation stages and the nonoverlapping subsets of lineage markers and fusion proteins, such as HLA class I molecules, expressed by various myeloid neoplasms [16–18]. HLA class I expression was normally expressed in CD34^+^ blasts and during myeloid differentiation in MDS patients; however, the copy-neutral loss of heterozygosity in the HLA region appeared to favor MDS progression to AML and selective downregulation of HLA class I enhanced *KIR AA* protection against different leukemia types [18–21].

Although no MDS patient showed total loss of HLA class I expression on CD34^+^ cells in peripheral blood, the HLA class I expression on dysplastic CD34^+^ cells was significantly lower in MDS patients than that in healthy controls (*P* = 0.002) [18]. A loss of heterozygosity in the *HLA* region may be as another possible immunoevasion mechanism in advanced cases of MDS [19]. These specific alterations can result in the inefficient presentation of immunodominant antigens to cytotoxic T lymphocytes but offer a necessary condition for NK cell-mediated immune response. The present study showed that the frequencies of both *HLA-Bw4* and *KIR3DL1-HLA-Bw4* significantly decreased in MDS patients. Because individual HLA allotypes belonging to the HLA-Bw4 group recognize distinct peptide motifs, it is unlikely that common peptide recognition explains the protection conferred by HLA-Bw4, which suggest that as the ligand for KIR3DL1, HLA-Bw4 protection against MDS probably has to do with NK cell-mediated immune response [22]. Base on NK cell education and “missing-self” recognition, we speculate that mature NK cell subsets that express inhibitory KIR3DL1 for self-HLA-Bw4 ligand may have greater activation potential when the inhibitory signal is interrupted through interactions with HLA-deficient cells such as dysplastic CD34^+^ cells, which may explain the candidate mechanism of a protective effect on MDS conferred by KIR3DL1-HLA-Bw4 in Chinese Southern Han. It has been well established that HLA-Bw4-80I serves as a much more efficient ligand for KIR3DL1 than HLA-Bw4-80T. Our results also revealed that HLA-Bw4-80I and KIR3DL1-HLA-Bw4-80I could confer a protective effect against MDS in Chinese Southern Han.

Owing to the low incidence of MDS in the general population reported as five new MDS diagnoses per 100,000 people [23], one limitation of this paper is that only 77 MDS patients have been included in the case group during the past ten years. In the follow-up research, it is necessary to expand the sample size of the MDS patient group and verify the function of NK cells in vitro.

## 5. Conclusions

The present study demonstrated that HLA-Bw4 (ligand for KIR3DL1) and the strong inhibitory KIR3DL1-HLA-Bw4 combination could confer a protective effect against MDS in Chinese Southern Han. The findings can provide valuable clues for better understanding pathogenesis of MDS in Chinese Southern Han.

## Figures and Tables

**Table 1:** Comparison analysis for *KIR* genes and *KIR AA* genotype between MDS patients and controls in Chinese Southern Han^∗^.

	Patients *n* (%)	Controls *n* (%)	OR (95% CI)	*P*
*2DL1*	77 (100)	739 (99.19)	/	/
*2DL2*	19 (24.68)	157 (21.07)	1.227 (0.710-2.121)	0.463
*2DL3*	76 (98.70)	733 (98.39)	1.244 (0.160-9.700)	1.000
*2DL5*	31 (40.26)	279 (37.45)	1.126 (0.697-1.817)	0.628
*2DS1*	27 (35.06)	238 (31.95)	1.150 (0.703-1.883)	0.577
*2DS2*	20 (25.97)	157 (21.07)	1.314 (0.767-2.253)	0.319
*2DS3*	19 (24.68)	130 (17.45)	1.550 (0.893-2.690)	0.117
*2DS4*	73 (94.81)	720 (96.64)	0.634 (0.215-1.871)	0.510
*2DS5*	17 (22.08)	173 (23.22)	0.937 (0.533-1.648)	0.821
*3DL1*	73 (94.81)	720 (96.64)	0.634 (0.215-1.871)	0.510
*3DS1*	26 (33.77)	247 (33.15)	1.028 (0.626-1.688)	0.914
*2DP1*	77 (100.00)	740 (99.33)	/	/
*KIR AA*	40 (51.95)	412 (55.30)	0.874 (0.546-1.398)	0.573

^∗^Note: the observed frequencies for the four framework *KIR* genes (*2DL4*, *3DL2*, *3DL3*, and *3DP1*) were all 100% in the patients and healthy controls. Thus, these framework *KIR* genes were not included in this table.

**Table 2:** Comparison analysis for *class I HLA* ligands and *KIR-HLA* combinations between MDS patients and controls.

	Patients *n* (%)	Controls *n* (%)	OR (95% CI)	*P*
HLA ligands				
*C1/C1 vs.*	52 (72.22)	520 (72.22)	1.000 (0.582-1.718)	1.000
*C1/C2*	20 (27.78)	200 (27.78)		
*C1/C1 vs.*	52 (92.86)	520 (97.38)	0.350 (0.111-1.102)	0.810
*C2/C2*	4 (7.14)	14 (2.62)		
*C1/C2 vs.*	20 (83.33)	200 (93.46)	0.350 (0.105-1.165)	0.092
*C2/C2*	4 (16.67)	14 (6.54)		
*C1+*^∗^*vs.*	72 (94.74)	720 (98.09)	0.350 (0.112-1.091)	0.079
*C2/C2*	4 (5.26)	14 (1.91)		
*C2+*^∗∗^*vs.*	24 (31.58)	214 (29.16)	1.121 (0.674-1.866)	0.659
*C1/C1*	52 (68.42)	520 (70.84)		
*Bw4 vs.*	48 (62.34)	547 (73.42)	0.599 (0.367-0.977)	0.038
Others	29 (37.66)	198 (26.58)		
*Bw4-80I vs.*	31 (40.26)	404 (54.23)	0.569 (0.353-0.917)	0.019
Others	46 (59.74)	341 (45.77)		
*Bw4-80T vs.*	27 (35.06)	246 (33.02)	1.095 (0.669-1.792)	0.717
Others	50 (64.94)	499 (66.98)		
*A3/A11*	49 (63.64)	450 (60.57)	1.139 (0.700-1.854)	0.599
Others	28 (36.36)	293 (39.43)		
KIR-HLA combinations				
*3DL1-Bw4 vs.*	46 (59.74)	528 (70.87)	0.610 (0.377-0.988)	0.043
Others	31 (40.26)	217 (29.13)		
*3DL1-Bw4-80I vs.*	29 (37.66)	392 (52.62)	0.544 (0.336-0.882)	0.012
Others	48 (62.34)	353 (47.38)		
*3DL1-Bw4-80T vs.*	22 (28.57)	238 (31.95)	0.852 (0.508-1.430)	0.544
Others	55 (71.43)	507 (68.05)		
*3DS1-Bw4 vs.*	16 (20.78)	166 (22.28)	0.915 (0.514-1.629)	0.762
Others	61 (79.22)	579 (77.72)		
*2DL1-C2 vs.*	24 (31.58)	213 (29.02)	1.129 (0.678-1.879)	0.641
Others	52 (68.42)	521 (70.98)		
*2DS1-C2 vs.*	8 (10.53)	70 (9.54)	1.116 (0.515-2.417)	0.781
Others	68 (89.47)	664 (90.46)		
*2DL2-C1 vs.*	18 (23.68)	154 (20.98)	1.169 (0.669-2.042)	0.583
Others	58 (76.32)	580 (79.02)		
*2DL3-C1 vs.*	71 (93.42)	709 (96.59)	0.501 (0.186-1.348)	0.190
Others	5 (6.58)	25 (3.41)		
*2DS2-C1 vs.*	18 (23.68)	154 (20.98)	1.169 (0.669-2.042)	0.583
Others	58 (76.32)	580 (79.02)		

Note: ^∗^*C1*+ includes *C1/C1* and *C1/C2* genotypes, ^∗∗^*C2*+ includes *C1/C2* and *C2/C2* genotypes.

## Data Availability

The data used to support the findings of the current study are provided within the article.

## Abbreviations

KIR: Killer cell immunoglobulin-like receptor
HLA: Human leucocyte antigen
MDS: Myelodysplastic syndromes
AML: Acute myeloid leukemia
ALL: Acute lymphoblastic leukemia
CML: Chronic myeloid leukemia
NHL: Non-Hodgkin's lymphoma.
